# Anti-Ageing Effects of *Sonchus oleraceus* L. (pūhā) Leaf Extracts on H_2_O_2_-Induced Cell Senescence

**DOI:** 10.3390/molecules20034548

**Published:** 2015-03-12

**Authors:** Zong-Quan Ou, Thomas Rades, Arlene McDowell

**Affiliations:** 1 School of Pharmacy, University of Otago, Dunedin 9056, New Zealand; E-Mail: zongquan.ou@otago.ac.nz; 2 Department of Pharmacy, University of Copenhagen, Copenhagen 2100, Denmark; E-Mail: thomas.rades@sund.ku.dk

**Keywords:** *Sonchus oleraceus*, antioxidants, leaf extracts, caffeic acid derivatives, CAA, senescence, anti-ageing

## Abstract

Antioxidants protect against damage from free radicals and are believed to slow the ageing process. Previously, we have reported the high antioxidant activity of 70% methanolic *Sonchus oleraceus* L. (Asteraceae) leaf extracts. We hypothesize that *S. oleraceus* extracts protect cells against H_2_O_2_-induced senescence by mediating oxidative stress. Premature senescence of young WI-38 cells was induced by application of H_2_O_2_. Cells were treated with *S. oleraceus* extracts before or after H_2_O_2_ stress. The senescence- associated β-galactosidase (SA-β-gal) activity was used to indicate cell senescence. *S. oleraceus* extracts showed higher cellular antioxidant activity than chlorogenic acid in WI-38 cells. *S. oleraceus* extracts suppressed H_2_O_2_ stress-induced premature senescence in a concentration-dependent manner. At 5 and 20 mg/mL, *S. oleraceus* extracts showed better or equivalent effects of reducing stress-induced premature senescence than the corresponding ascorbic acid treatments. These findings indicate the potential of *S. oleraceus* extracts to be formulated as an anti-ageing agent.

## 1. Introduction

Ageing or senescence is a complex and inevitable biological process, which is not only attributed to individual genetic variation but also to external factors such as environmental conditions, nutrition, alcohol and diseases [1,2,3,4]. There are three theories that have been proposed to explain ageing: genetic, neuroendocrine and damage-accumulation theories, which are all important and interrelated [5]. Of these theories, the most widely accepted is the free radical theory that ageing and its related diseases result from accumulated oxidative damage to cell constituents and tissues caused by excessive exposure to free radicals [6]. Reactive species are generated during normal cell metabolism [7] or to protect humans from immediate death from infections [8]. These reactive species can cause lipid peroxidation, denature proteins and fragment DNA due to oxidative stress, and further increase the mutation rate, especially when the antioxidant defenses cannot remove the excess free radicals [5,7,8]. Increasing oxidative stress is commonly associated with ageing and age-related diseases [9].

Antioxidants, which mediate the imbalance between intracellular antioxidant defenses and oxidative damage by reducing the reactive oxygen species (ROS) levels, are believed to be able to reduce stress-induced premature senescence or slow down replicative senescence. Investigations with ascorbic acid and vitamin E [10,11,12,13], or plant-derived antioxidants [14,15,16,17] confirm this claim. Due to the reported carcinogenesis and hepatoxicity of synthetic antioxidants [18], antioxidants from natural origins are preferable in applications for medications and food additives. Both normal human fibroblasts and H_2_O_2_-stressed fibroblasts experienced an extended lifespan in the presence of cyanidin, the most prevalent anthocyanin in plants [19]. Likewise in *in vivo* studies, protocatechuic acid from *Alpinia oxyphylla* fruits successfully enhanced endogenous antioxidant levels and therefore their activity, and down-regulated malondialdehyde (a biomarker of ageing) in aged rats [14].

Cellular senescence can occur due to failure in cell proliferation, or exposure to a variety of cellular stresses, such as oxidizing or DNA damaging agents, or expression of activated oncogenes [3,20]. A variety of cellular senescence features have been identified, amongst which are cellular morphology [2,21], telomere length [2,22], gene expression [23] and senescence-associated β-galactosidase (SA-β-gal) activity [24,25]. During ageing, senescence-related genes can be over-expressed to induce senescent morphogenesis [23]. Many senescence-associated, over-expressed proteins can be utilized as biomarkers, such as transforming growth factor-β1, ICFBP-3 mRNA, p16 tumor suppressor protein and β-galactosidase [23,24,25]. β-Galactosidase is a collective name for enzymes that cleave non-reducing β-d-galactose residues from glycoproteins, sphingolipids and keratin sulphate in β-d-galactosides [20]. In senescent cells, the over-expression of β-galactosidase enables the detection of β-galactosidase activity at sub-optimal pH [3,24]. This enzyme activity is not detectable in actively proliferating cells. There are two main methods to measure the SA-β-gal activity [3]. It can be cytochemically or histochemically detected using the chromogenic substrate 5-bromo-4-chloro-3-indoyl β-d-galactopyranoside. SA-β-gal positive (blue-stained) cells are manually counted and expressed as the percentage of total cell population [24]. The cytochemical assay is simple and allows detection in tissue samples. On the other hand, it is subjective and the procedure is time-consuming. The second method is fluorescence-based using the fluorogenic substrates 5-dodecanoylaminofluorescein-di-β-d-galactopyranoside (C_12_FDG) or fluorescein-di-β-d-galacto-pyranoside (FDG) lysosome alkalinizaiton. SA-β-gal positive activity can be detected and quantified using a flow cytometer, microfluidics analyzer or fluorescence microscope [3]. The fluorescence-based methods permit quantitative measurement of single cells in the population. Compared with cytochemical assays, fluorescent assays are more sensitive and accurate with higher throughput.

*Sonchus oleraceus* L. (family Asteraceae) is well-known for its high content of antioxidants and antioxidant activity [26,27,28,29,30]. In New Zealand, it is commonly known as pūhā, where it is a dietary and traditional medicinal plant in Māori culture. We have previously identified three major antioxidants in methanolic leaf extracts of *S. oleraceus*: caftaric acid, chlorogenic acid and chicoric acid, with chicoric acid having the highest concentration [31]. To the authors’ knowledge, there is no report of the potential anti-ageing effects of *S. oleraceus* leaf extracts, neither *in vitro* nor *in vivo*. We have previously shown that *S. oleraceus* leaf extracts are absorbed into cells *in vitro* and can exert an antioxidant effect, but it remains unclear if the antioxidants in *S. oleraceus* leaves are beneficial for cells in combating oxidative senescence. Demonstration of antioxidant activity in healthy cells is a useful screening method for the bioactivity of novel compounds, however to be useful as therapeutic agents, these compounds must show effects in a disease state. Herein, we investigated the effects of *S. oleraceus* leaf extracts on H_2_O_2_-stressed human lung fibroblasts. SA-β-gal activity was used as the biomarker indicating premature senescence of the cells.

## 2. Results and Discussion

### 2.1. Cell Viability

**Figure 1:**
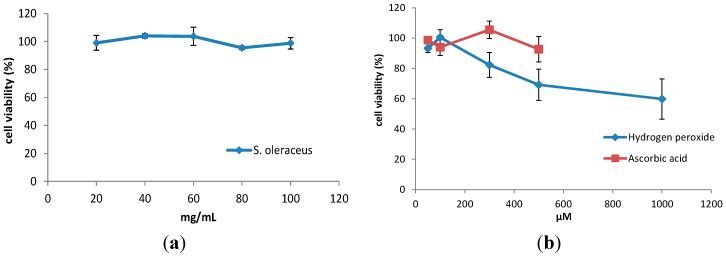
Viability of WI-38 cells after treatment with (**a**) various concentrations of *S. oleraceus* leaf extracts from 20 to 100 mg/mL and (**b**) 50–1000 μM H_2_O_2_ or 50–500 μM ascorbic acid for 3 h. Data represent triplicate treatments and are expressed as mean ± SD (*n* = 3).

WI-38 cells retained greater than 90% viability after 3 h incubation with leaf extracts from *S. oleraceus* (Figure 1a). It is assumed that concentrations of the leaf extract lower than 20 mg/mL would also be non-toxic. With addition of H_2_O_2_, cell viability decreased with increasing concentration (Figure 1b). Therefore, a concentration of 100 µM or lower H_2_O_2_ was chosen for further experiments.

### 2.2. Cellular Antioxidant Activity (CAA) Assay

**Figure 2:**
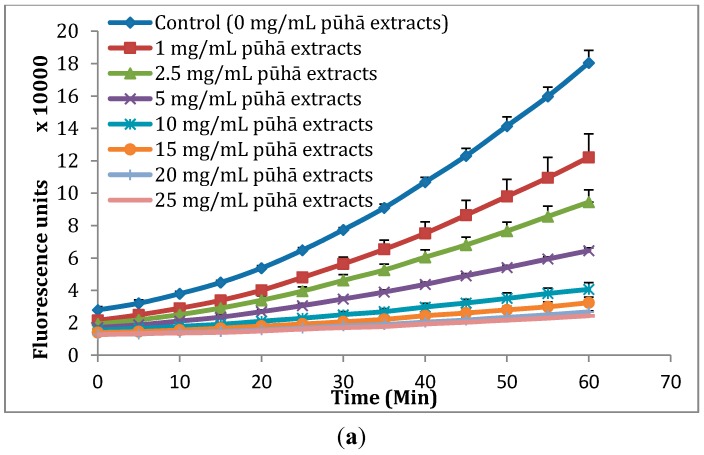

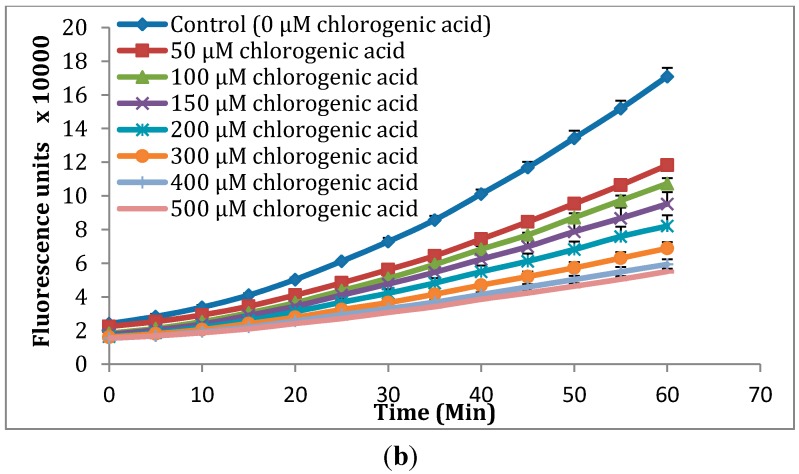
Peroxyl radical-induced oxidation of DCFH to DCF in WI-38 cells and inhibition of oxidation by (**a**) *S. oleraceus* leaf extracts and (**b**) chlorogenic acid with time. The fluorescence intensity indicates the oxidation of DCFH. The curves shown in each graph are from a single experiment (mean + SD, *n* = 3).

**Figure 3:**
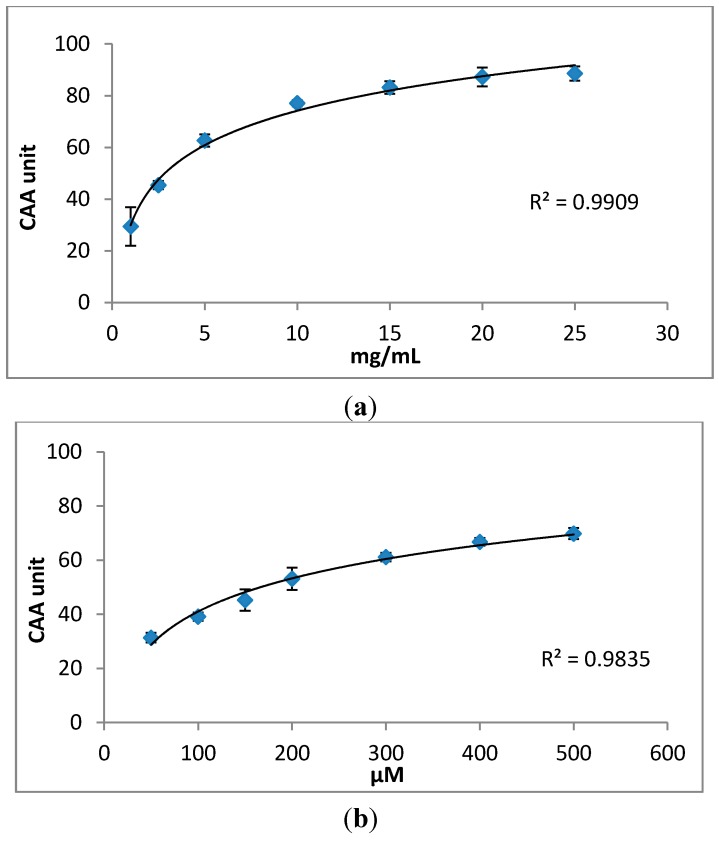
Dose–response curves for inhibition of peroxyl radical-induced DCFH oxidation by (**a**) *S. oleraceus* leaf extracts and (**b**) chlorogenic acid. The data were expressed as CAA unit indicating the intra-cellular antioxidant activity of samples. The curves shown are each from a single experiment (mean ± SD, *n* = 3).

The CAA assay quantifies the antioxidant activity by measuring the ability of applied compounds to prevent oxidation in cells. In the CAA assay, DCFH-DA is converted to fluorescent DCF by peroxyl radicals [32]. The fluorescence intensity is proportional to the ROS concentration in the cells. The curves in the Figure 2 indicate the formation of the fluorescent DCF over 60 min. The flatter the curve, the less DCFH is oxidised. Using the CAA assay we found that the oxidation of DCFH to DCF was reduced by *S. oleraceus* leaf extracts in a dose-dependent manner within the concentration range of 1–25 mg/mL (Figure 2a). Intracellular ROS due to normal cell metabolism also would cause the oxidation of DCFH. However, the present results are processed data after subtracting a blank (oxidation of DCFH by normal cell metabolism) from raw data. Chlorogenic acid also decreased the formation of fluorescence with increasing concentration from 50 to 500 μM (Figure 2b). For the two other major compounds identified in *S. oleraceus* leaf extracts, caftaric acid and chicoric acid, the fluorescence was completely quenched (data not shown). Comparing *S. oleraceus* extracts with chlorogenic acid, *S. oleraceus* leaf extracts showed much higher activity with an EC_50_ value of 2.85 mg/mL (50.16 μM equivalent of total concentration of the three key antioxidants) than that of chlorogenic acid with an EC_50_ value of 166.34 μM. 1 mg/mL *S. oleraceus* leaf extracts and 50 μM chlorogenic acid showed similar inhibition of the fluorescence (Figure 2). This also can be confirmed by the CAA units obtained from the CAA assay (Figure 3). The CAA unit from the CAA assay indicates the intra-cellular activity of the samples. 1 mg/mL of *S. oleraceus* leaf extracts (containing 17.6 μM of the three key active compounds in total) exhibited a trend of higher activity, with 29.44 ± 7.44 CAA units, than that of 50 μM chlorogenic acid (31.34 ± 1.79 CAA units) (Figure 3). Within the concentration ranges studied, *S. oleraceus* leaf extracts showed higher potency than chlorogenic acid alone.

### 2.3. Effects of S. oleraceus Leaf Extracts on Stress-Induced Premature Senescence in WI-38 Cells

#### 2.3.1. Protective Effect

**Figure 4:**
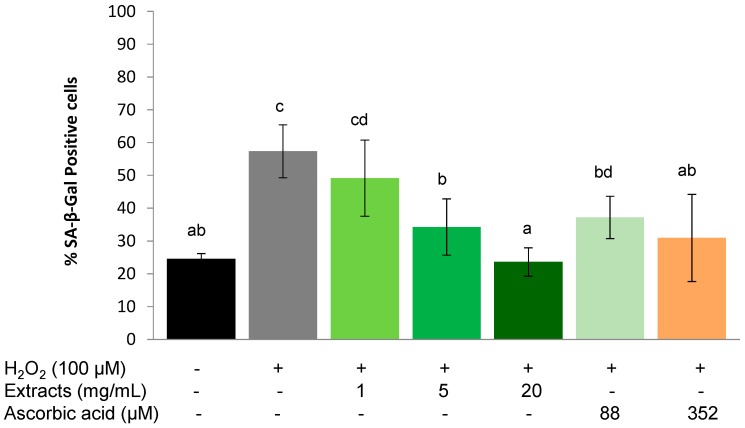
Protective effect of *S. oleraceus* leaf extracts on H_2_O_2_ treated WI-38 cells. Cells were treated with 1, 5 or 20 mg/mL *S. oleraceus* leaf extracts or 88 or 352 µM ascorbic acid for 1 h before being stressed with 100 µM H_2_O_2_. Data are shown as percentage of SA-β-Gal positive cells (mean ± SD, *n* = 6). Bars with no letters in common are significantly different (*p* < 0.05).

The SA-β-Gal positive cell population increased significantly (*p* < 0.01) from 24.6 ± 1.6 to 57.4% ± 8.1% after being stressed with 100 µM H_2_O_2_ (Figure 4). There was a concentration-dependent effect of the *S. oleraceus* leaf extracts on the proportion of SA-β-Gal positive cells (Figure 4), and a trend for pre-treatment with 1 mg/mL *S. oleraceus* leaf extracts to reduce the percentage of SA-β-Gal positive cells. At 5 mg/mL, *S. oleraceus* leaf extracts reduced two thirds of H_2_O_2_ stress-induced SA-β-Gal activity. Stress-induced premature senescence was completely suppressed by 20 mg/mL *S. oleraceus* leaf extracts and was equivalent to the control group that did not receive the H_2_O_2_ stress (Figure 4). Compared to cells with corresponding ascorbic acid treatments (88 and 352 µM), 5 and 20 mg/mL *S. oleraceus* leaf extracts had a tendency to show better protective effects, but these data were not statistically significant.

#### 2.3.2. Recovery Effect

A similar trend as for the protective effect was observed for the ability of *S. oleraceus* extracts to assist in the recovery of WI-38 cells from the application of exogenous stress. 1 h treatment with 100 µM H_2_O_2_ significantly (*p* < 0.01) increased SA-β-Gal positive cells from 21.55% ± 4.12% to 33.40% ± 4.25%. However, this increase was lower than that of H_2_O_2_ treatment in the protective experiment (Figure 4). *S. oleraceus* extracts (except 1 mg/mL) and ascorbic acid reduced the SA-β-Gal activity caused by H_2_O_2_, and showed a concentration-dependent effect. When the concentration of *S. oleraceus* extracts was increased to 5 or 20 mg/mL, SA-β-Gal activity induced by H_2_O_2_ was significantly (*p* < 0.05) reduced. 

**Figure 5:**
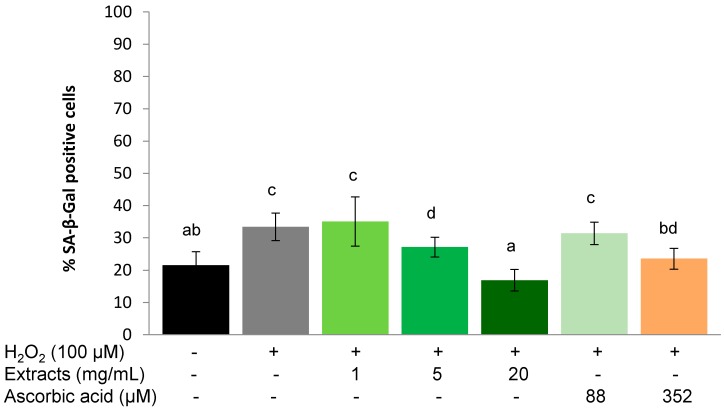
Recovery effect of *S. oleraceus* leaf extracts on H_2_O_2_ treated WI-38 cells. Cells were treated with 1, 5 or 20 mg/mL *S. oleraceus* leaf extracts or 88 or 352 µM ascorbic acid for 1 h following a 1 h incubation with 100 µM H_2_O_2_ as exogenous stress. Data are shown as percentage of SA-β-Gal positive cells (mean ± SD, *n* = 6). Bars with no letters in common are significantly different (*p* < 0.05).

Furthermore, 5 and 20 mg/mL *S. oleraceus* extracts showed significantly (*p* < 0.05) better recovery ability than the corresponding ascorbic acid treatments, although 352 µM ascorbic acid also significantly reduced the SA-β-Gal activity (*p* < 0.01) compared to H_2_O_2_ stress (Figure 5).

**Figure 6:**
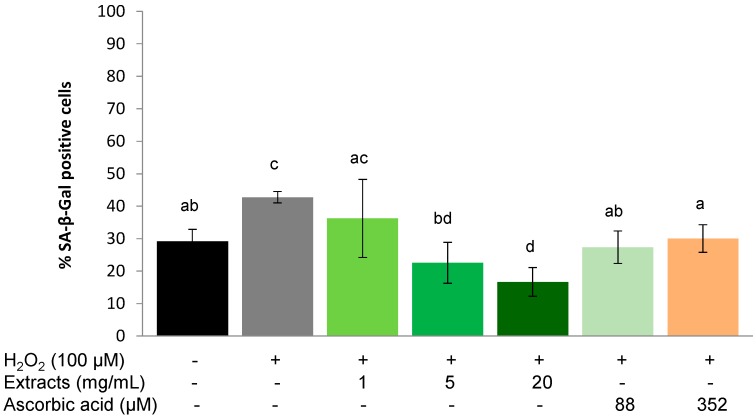
Protective effect of *S. oleraceus* leaf extracts on WI-38 cells chronically treated with H_2_O_2_. Cells were treated with 1, 5 or 20 mg/mL *S. oleraceus* leaf extracts or 88 or 352 µM ascorbic acid for 1 h at day 1 before being stressed with 30 µM H_2_O_2_ 1 h per day for five consecutive days. Data are shown as percentage of SA-β-Gal positive cells (mean ± SD, *n* = 6). Bars with no letters in common are significantly different (*p* < 0.05).

#### 2.3.3. Protective Effect on Chronic Stress

SA-β-Gal positive cells increased significantly (p < 0.01) after consecutive stress of 30 µM of H_2_O_2_ each day for 5 days (Figure 6). 1 h treatment with *S. oleraceus* leaf extracts (except 1 mg/mL) or ascorbic acid significantly reduced the SA-β-Gal positive cell population, indicating a sustained protective effect of one acute dose of these antioxidants from repeated exposure to low H_2_O_2_ levels (Figure 6). In line with the acute protective and recovery effects, a concentration-dependent effect of *S. oleraceus* leaf extracts was also observed (Figure 6). The highest concentration of *S. oleraceus* leaf extracts tested was also more effective at reducing SA-β-Gal activity than the equivalent ascorbic acid treatment.

### 2.4. Discussion

There is growing interest in the use of natural antioxidants for human health benefits or even as food preservatives due to the safety issues raised by the use of synthetic antioxidants [33]. Many natural products have been reported to have anti-ageing activity, including herbal extracts such as *Rhodiola rosea* (Crassulaceae), *Rosa damascene* (Rosaceae), *Damnacanthus officinarum* (Rubiaceae), spinach leaf and green tea extracts [34]. *S. oleraceus*, a traditional medicinal plant worldwide, is well-known for its high antioxidant content. It is reported that *S. oleraceus* leaf extracts are non-cytotoxic on HepG2 cells below a concentration of 100 mg/mL [30]. To our knowledge, there is no report on the application of *S. oleraceus* leaf extracts on normal human diploid cells. In the present study, no cytotoxicity of *S. oleraceus* leaf extracts up to 100 mg/mL was observed in WI-38 cells (Figure 1).

The measurement of antioxidant activity is an important part of screening plant extracts that have the potential to be developed into supplements. Previously, we reported high antioxidant activity of *S. oleraceus* leaf extracts determined by the DPPH free radical assay [30,35]. However, a high antioxidant activity as measured by *in vitro* chemical assays does not necessarily correlate to relevant *in vivo* activity because of the limited predictability in complex biological systems. For instance, extracts from plum fruit had the same chemical antioxidant activity using the ORAC assay as blackberry extracts, but significantly lower cellular activity than blackberry extracts [36]. Nevertheless, some studies do show some correlations between chemical and cellular measurements of antioxidant activity of plant samples [37,38]. Cellular antioxidant activity is an important and useful parameter that indicates antioxidant activity of samples, and is more biologically representative compared to test tube chemical antioxidant activity measurements. The CAA assay developed by Wolfe and Liu [32] utilizes peroxyl radicals to assess antioxidant activity and takes into account cellular uptake, distribution and metabolism by incubating cells with antioxidants prior to exogenous application of oxidative stress. Using this assay in the present study, it is observed that *S. oleraceus* leaf extracts were absorbed by and exhibited antioxidant activity in WI-38 cells with an EC_50_ value of 2.85 mg/mL (Figure 2). This activity is lower than the reported value for blueberry (10.81 mg/mL) [32]. This indicates the high potency of *S. oleraceus* extracts, with values also higher than a range of vegetables [39], including broccoli, lettuce and tomatoe; and a range of other fruits [36], including apple and kiwifruit. 1 mg/mL *S. oleraceus* leaf extracts with 17.6 µM of the total key antioxidants had a comparably high activity to that of 50 µM chlorogenic acid, suggesting that the antioxidant extract matrix is more effective than chlorogenic acid alone. It is also possible that chlorogenic acid is not the major contributor to the cellular activity of *S. oleraceus* leaf extract.

Ageing is inevitable, but processes that are induced by external factors can be slowed by down-regulating oxidative stress in the body, for instance, by supplementing with antioxidants. Hwang *et al.* [13] demonstrated that ascorbic acid extends the replicative life span of human embryonic fibroblasts by decreasing cellular ROS to reduce mitochondrial and DNA damage. In the present study, ascorbic acid and *S. oleraceus* extracts containing an antioxidant mixture also exhibited an anti-ageing effect by reducing stress-induced premature senescence (Figure 4, Figure 5, and Figure 6). However, long-term ascorbic acid supplementation has shown conflicting effects in animal studies. A diet of an antioxidant combination showed no increase in the life span of rats [40]. The authors proposed that compensatory reductions in endogenous protection mechanisms might be involved when high dietary doses of vitamin C were administered. For this reason, the concentration of ascorbic acid used for the present study has been carefully chosen. Cells with stress-induced, premature senescence share similar characteristics to those with replicative senescence, such as morphology, cell cycle regulation, increased ROS generation and SA-β-gal activity [2]. Stress-induced premature senescence can be induced by various sub-lethal stresses including H_2_O_2_, hyperoxia, UV radication or *tert*-butylhydroperoxide [2,9,41]. Human fibroblasts undergo finite proliferation before irreversible growth arrest *in vitro* [42]. Human diploid fibroblasts have become a classical model for studying cellular ageing and identifying ageing-related changes in human cells. Based on these features, H_2_O_2_ stressed human fibroblast cells are considered a suitable model to assess effects of anti-ageing agents [9,19,43]. SA-β-gal activity is one of the most popular biomarkers for cell senescence [44,45]. The SA-β-gal activity in the present study increased significantly in the presence of H_2_O_2_, suggesting that cells experienced senescence. Both *S. oleraceus* extracts and ascorbic acid suppressed SA-β-gal activity caused by H_2_O_2_, indicating the anti-senescence properties of *S. oleraceus* extracts and ascorbic acid. 88 and 352 µM ascorbic acid (equivalent to 5 and 20 mg/mL *S. oleraceus* extracts, respectively) did not show better ability than the corresponding *S. oleraceus* extracts. This implied that *S. oleraceus* extracts at a concentration of 20 mg/mL and above might have better ability of protecting or recovering stress-induced premature senescence than ascorbic acid.

From the above, it is clear that *S. oleraceus* leaf extracts can protect human cells from stress-induced senescence. However, if this effect is long lasting or if frequent treatments are required to maintain this effect remain unknown. In view of this, a chronic stress experiment was carried out to further explore the anti-ageing effect of *S. oleraceus* leaf extracts. The results demonstrated a similar effect pattern as that of acute stresses (Figure 6). This suggested that *S. oleraceus* extracts can not only assist cells to recover from acute H_2_O_2_ stress, but can also protect cells from chronic stress applied for five consecutive days as tested in the present study. Similar to the present results, a mixture of cocoa and green tea extracts, rich in polyphenols, and vitamin E significantly reduced UV stress-induced premature senescence from 51.67% to 38.81% in human dermal fibroblasts [46]. Oxidative DNA damage has been demonstrated to be proportional to the age of WI-38 human fibroblast cells [9] and was proposed to be another biomarker for cellular senescence. H_2_O_2_-induced DNA damage [43] thus showed senescent-like growth arrest [47] in human fibroblasts. In accordance with previous studies, despite the use of a different biomarker in the present study, H_2_O_2_ significantly increased stress-induced premature senescence (Figure 4, Figure 5, and Figure 6). It is reported that antioxidant treatments counteracted the effect of stressors, including H_2_O_2_, and restored DNA synthesis [48]; and therefore can protect the cells from premature ageing. Further studies are needed to understand how *S. oleraceus* leaf extracts mediate the effects of the external stress factors on cells. For example, utilizing the comet assay [49] to characterize the extent of DNA damage. An understanding of the cellular components that are damaged by application of stress would help to define which of the cellular processes are involved during recovery from stress.

The protective and recovery ability of *S. oleraceus* extracts against H_2_O_2_ stress-induced premature senescence in the present study was encouraging for the use of *S. oleraceus* leaves as a potent anti-ageing, nutritional supplement. In the development of oral supplements, it is imperative to characterize the *in vivo* stability of bioactive components in the gastrointestinal tract environment. Further, to ensure the therapeutic effect of the *S. oleraceus* leaf extracts following oral administration, formulation strategies that increase the *in vivo* stability, absorption and bioavailability should be investigated.

## 3. Experimental Section

### 3.1. Plant Material

*S. oleraceus* seeds were collected from plants growing wild in Oamaru, New Zealand (45°05.346' S, 170°58.861' E) and identified by Mr John Steel, a botanist in the Department of Botany at the University of Otago, New Zealand. Voucher specimens (OTA 061166 and OTA 061167) are lodged at the Otago Regional Herbarium in the Department of Botany at the University of Otago, New Zealand. Seeds were germinated in a 50:50 potting compost:sand mix and grown for three weeks in a greenhouse, and the seedlings transplanted into 1 L pots in the same soil mix as above. Tap water was supplied every two days. Artificial light was provided 24 h a day in addition to normal natural daylight. The temperature inside the greenhouse was maintained at 25 °C. Fully expanded mature leaves from the middle of eight-week-old *S. oleraceus* plants were harvested and freeze-dried before being blended into a powder.

### 3.2. Preparation of Leaf Extracts

Freeze-dried *S. oleraceus* leaf powder was extracted with 70% methanol (*v*/*v*) to obtain a 10% (fresh *w*/*v*) mixture as described by Ou *et al.* [31]. After stirring for approximately 0.5 h at room temperature, the extraction mixture was centrifuged at 9700 *g* for 10 min. Solvent was removed by centrifugal evaporation (SVC-200H SpeedVac^®^ Concentrator, Savant, Farmingdale, NY, USA). The dried extracts were stored at −20 °C before further analysis.

### 3.3. Preparation of Extract Samples and Chemical Solutions

*S. oleraceus* extracts were prepared from freeze-dried leaf powder as described above. Chlorogenic acid (99% GR) (Acros Organics, Geel, Belgium) and ascorbic acid (Sigma^®^, St. Louis, MO, USA) and extract solutions were prepared immediately prior to use. For cytotxicity measurements, methylene blue stain solution consisted of 98% HBSS (*v*/*v*), 0.5% glutaraldehyde and 0.6% methylene blue (*w*/*v*) (Koch-Light laboratories Ltd., UK). Elution solution was made of 49% phosphate buffered saline solution (PBS) (Gibco^®^, Grand Island, NY, USA), 50% ethanol and 1% acetic acid (*v*/*v*) (BDH Aristar^®^, Edmonton, AB, Canada).

For the Cellular Antioxidant Activity (CAA) assay, a 20 mM stock solution of 2',7'-dichloro-fluorescin diacetate (DCFH-DA) (Sigma^®^, Rehovot, Israel) in methanol was prepared and stored at −20 °C. A 200 mM 2,2'-azobis(2-methylpropionamidine) dihydrochloride (ABAP) (Sigma^®^) stock solution in Hank’s balanced salt solution (HBSS) (Sigma^®^) was prepared, and aliquots were stored at −20 °C. Chlorogenic acid was dissolved in purified water before further dilution in treatment medium (Basal Medium Eagle (BME) (Gibco^®^) with 2 mM GlutaMAX (Gibco^®^) and 25 mM 2-[4-(2-hydroxyethyl)piperazin-1-yl]ethanesulfonic acid (HEPES) (Sigma^®^). Dried *S. oleraceus* extracts were re-dissolved and further diluted to required working concentrations in treatment medium.

For the ageing study, dried leaf extracts were re-dissolved in treatment media resulting in a concentration of 100 mg/mL (fresh weight). 1 mM stock solution of ascorbic acid was prepared in purified ionized water immediately prior to experiments. The filtered *S. oleraceus* extracts and ascorbic acid stock solution were further diluted in treatment media to working concentrations. 30% H_2_O_2_ was diluted to required working concentrations using treatment media. Bafilomycin A1 (>95%) (Merck Sydney, Australia) was dissolved in dimethyl sulfoxide (DMSO) (Sigma^®^, Lyon, France) to a final concentration of 0.1 mM. For experimental application, the bafilomycin A1 stock was diluted to 100 nM in cBME (BME supplemented with 10% Fetal bovine serum (FBS) (Gibco^®^, Auckland, New Zealand), 2 mM GlutaMAX plus 25 mM HEPES, pH 7.2). 5-Dodecanoylaminofluorescein Di-β-d-galacto-pyranoside (C_12_FDG) (Setareh Biotech, Eugene, OR, USA) stock solution was also prepared in DMSO to a final concentration of 20 mM. The 20 mM stock solution of C_12_FDG was diluted 1:10 with fresh cBME to make a 2 mM working solution immediately before its addition into the cells. The final concentration of C_12_FDG obtained in cells was 33 μM. Fluorescence-activated cell sorting (FACS) buffer was prepared by dissolved 1% (*w*/*v*) bovine serum albumin (Gibco^®^, New Zealand) and 0.01% (*w*/*v*) sodium azide (Sigma^®^) in PBS.

### 3.4. Cell Culture

WI-38 human lung diploid fibroblast cells (Coriell Cell Repositories, Camden, NJ, USA) were cultured in cBME at 37 °C with 5% CO_2_. The medium was changed every 3–4 days. Cells used for this study were at population doubling level (PDL) 19–27. The PDL of the cell culture was determined as follows: current PDL = last PDL + 3.32 × Log (harvested cell population/seeded cell population).

### 3.5. Cell Viability

Cell viability was measured using the method of Wolfe *et al.* [32] with modifications. WI-38 cells were seeded in a 96-well plate at a density of 5 × 10^4^ cells per cm^2^ and cultured in cBME. After 24 h incubation, cells were treated with various concentrations of *S. oleraceus* extracts or ascorbic acid in treatment media for 3 h or 24 h, or treated with H_2_O_2_ only for 2 h. Cells were then washed with 100 µL HBSS and 50 µL methylene blue staining solution was added into each well. After another hour of incubation at 37 °C, the dye was washed off using fresh deionized water and the plate was air-dried following elution.

### 3.6. Cellular Antioxidant Activity (CAA) Assay

The cellular antioxidant activity was measured using a modified method from Wolf *et al.* [32]. WI-38 cells were seeded at 5 × 10^4^ cells per cm^2^ on a 96-well plate in 100 µL of cBME, excluding the outside wells of the plate. After incubation for 24 h, the cBME was removed and the wells were washed with PBS. Triplicate wells were treated with 100 µL of *S. oleraceus* extracts and 25 µM DCFH-DA dissolved in treatment media and incubated for 1 h. Cells were then washed with 100 µL PBS, before 600 µM ABAP was applied to the cells in 100 µL of HBSS. Fluorescence was read with emission at 538 nm and excitation at 485 nm every 5 min for 1 h at 37 °C (POLARstar Omega microplate reader, BMG Labtech, Cary, NC, USA).

### 3.7. Quantification of CAA

After blank subtraction from the fluorescence readings, the area under the curve of fluorescence *versus* time was integrated to calculate the CAA unit as:

CAA unit = 100 − (∫SA/∫CA) × 100

where ∫SA and ∫CA are the integrated areas under the curve of sample and control, respectively [32]. The median effective concentration (EC_50_) was determined for the *S. oleraceus* extracts and chlorogenic acid from the dose-response curve where the CAA unit is equal to 50.

### 3.8. Stress-Induced Premature Senescence in WI-38 Cells

#### 3.8.1. Protective Effect of S. oleraceus Leaf Extracts

To investigate if *S. oleraceus* leaf extracts can protect human cells from exposure to oxidative stress, WI-38 cells were seeded at a density of 5 × 10^4^ cells per cm^2^ in a 24-well plate. After 24 h incubation, cells were treated with 1, 5 or 20 mg/mL (fresh weight) *S. oleraceus* leaf extracts or 88 or 352 µM ascorbic acid in treatment media for 1 h. 88 or 352 µM ascorbic acid were chosen as the equivalent concentrations to the total concentrations of the three key antioxidants in 5 and 20 mg/mL extracts, respectively. Then cells were washed with HBSS before being stressed with 100 µM H_2_O_2_ in treatment media for another 1 h. Fresh cBME was added after the cells were washed with HBSS. Cells without any treatment were used as unstained controls. Cells with H_2_O_2_ treatment only were used as positive controls. After 48 h rest from stress, cells were treated for FACS measurement as detailed below.

#### 3.8.2. Recovery Ability of *S. oleraceus* Leaf Extracts

The recovery ability of *S. oleraceus* leaf extracts was conducted in the same way as the protective effect study described above with one modification: WI-38 cells were stressed with 100 µM H_2_O_2_ for 1 h before being treated with 1, 5 or 20 mg/mL (fresh weight) *S. oleraceus* leaf extracts or 88 or 352 µM ascorbic acid.

#### 3.8.3. Protective Effect from Chronic Stress

WI-38 cells were treated for 1 h with 1, 5 or 20 mg/mL (fresh weight) *S. oleraceus* leaf extracts or 80 or 352 µM ascorbic acid before chronic stress. Chronic stress was applied by adding 30 µM of H_2_O_2_ for 1 h per day 5 days after a single treatment of *S. oleraceus* leaf extracts or ascorbic acid. General procedures were otherwise the same as described above in the protective effect experiment.

### 3.9. Analysis of Senescence-Associated β-Galactosidase (SA-β-gal) Activity in WI-38 Cells

The SA-β-gal activity was determined as described by Debacq-Chainiaux *et al.* [3] with modifications. After 48 h rest from stress, cells in 24-well plates were treated with 100 nM bafilomycin A1 for 1 h in fresh cBME (0.5 mL) at 37 °C to induce lysosomal alkalinisation to facilitate measurement of the SA-β-gal activity at pH 6. 8.25 µL of 2 mM C_12_FDG working solution (C_12_FDG is a fluorogenic membrane-permeable substrate of β-galactosidase) was added to the media to give a final concentration of approximately 33 µM and the incubation was continued for another 1 h. The solution was removed and the cell monolayers were washed twice for approximately 30 s with 0.5 mL of PBS at room temperature. Cells were then trypsinized (TrypLE™) (Gibco^®^) and harvested followed by centrifugation at 100 *g* for 10 min at 4 °C. Cells were re-suspended in ice-cold FACS buffer and analysed immediately in a FACScalibur flow cytometer (FACSCanto II, Becton Dickinson, Franklin Lakes, NJ, USA). FlowJo software (Version 9.5.2, TreeStar, Inc., Ashland, OR, USA) was used for data analysis. Propidium iodide (BD Biosciences, San Jose, CA, USA) was used as dead/living cell indicator for all the cell samples.

### 3.10. Statistics

Either a Student’s *t-*test using Microsoft Excel (Version 2007) or analysis of variance with post-hoc Tukey’s multiple comparisons using SPSS Statistics from IBM (International Business Machines Corp., Armonk, NY, USA, Version 20) were used to establish significant differences between the groups. *p*-values < 0.05 were considered to be significant.

## 4. Conclusions

In summary, our results demonstrate that *S. oleraceus* extracts possessed high cellular antioxidant activity, compared to chlorogenic acid or other plant samples reported in literature. H_2_O_2_ stress-induced premature senescence can be significantly suppressed by *S. oleraceus* extracts at 5 mg/mL or above, and this anti-ageing effect is concentration-dependent. When compared to the corresponding ascorbic acid treatments, 5 and 20 mg/mL *S. oleraceus* extracts showed better or equivalent effects. These data suggest the therapeutic potential of *S. oleraceus* extracts as an anti-ageing agent.
